# Experimental Investigation and Numerical Simulation of a Self-Wiping Corotating Parallel Octa-Screw Extruder

**DOI:** 10.3390/polym14061201

**Published:** 2022-03-16

**Authors:** Cheng-Ying Liu, Shota Mikoshiba, Yutaka Kobayashi, Akira Ishigami, Daisuke Yorifuji, Shin-ichiro Tanifuji, Hiroshi Ito

**Affiliations:** 1 Graduate School of Organic Materials Science, Yamagata University, 4-3-16 Jonan, Yonezawa, Yamagata 992-8510, Japan; tks70095@st.yamagata-u.ac.jp (C.-Y.L.); s135sibatennislove@gmail.com (S.M.); akira.ishigami@yz.yamagata-u.ac.jp (A.I.); 2 Research Center for Green Materials and Advanced Processing, Yamagata University, 4-3-16 Jonan, Yonezawa, Yamagata 992-8510, Japan; kobayashi.y@yz.yamagata-u.ac.jp; 3 HASL, Shakuji machi, Nerimaku, Tokyo 177-0041, Japan; yorifuji@hasl.co.jp (D.Y.); tanifuji@hasl.co.jp (S.-i.T.)

**Keywords:** octa-screw extrusion, experimental investigation, numerical simulation

## Abstract

An octa-screw extruder (OSE) is equipment for pelletizing, blending, and mixing polymers and composites. In this study, the degree of resin filling, residence time distribution (RTD) of molten resin, and temperature profile in the octa-screw extruder were evaluated both experimentally and numerically. An intermeshing corotating parallel octa-screw kneading extruder was used for the experiments. For the comparison study, the results obtained from this extruder were compared with the twin-screw extruder. High-density polyethylene was selected as the material for extrusion. Meanwhile, a numerical code, based on a 2.5 D finite element method derived from the Hele–Shaw flow model, was developed to simulate the octa-screw extrusion process. The empirical outcomes suggest that octa-screw extrusion exhibited a narrower RTD of the molten resin compared with the twin-screw extrusion, suggesting better extrudate quality. The octa-screw extrusion also showed a lower temperature profile than twin-screw extrusion. The results of the simulation were also found to be in good agreement with experimental measurements. Experimental and numerical investigations of an OSE enable detailed comprehension and visualization of resin distribution in the entire length of the OSE, thus providing advantages in terms of process optimization.

## 1. Introduction

The self-wiping corotating parallel octa-screw extruder (OSE) is a high-efficiency polymer-processing machine for the pelletizing, blending, alloying, compounding, devolatilizing, chemical reacting, and die forming of molten polymers [1]. During the operation, the rotary motion of the parallel screws conveys the materials entering from the hopper into the feed throat. The mechanical shear from the screws and thermal dissipation energy from the barrel converts the solid polymer into the melt, which is pushed out of the die. To date, although twin-screw extrusions have become an important part of polymer-processing technologies and have been well studied through the years [2,3,4,5,6], the octa-screw has yet to be discovered. Compared to twin-screw extrusion processing, octa-screw extrusion processing provides outstanding mixing capability and greater throughput and exhibits a high potential for product commercialization. Venting and side feeding are also feasible for the octa-screw extrusion. However, octa-screw extrusion is mechanically more complex and less robust and has a higher cost relative to the twin-screw extrusion process.

The flow of material in OSEs is complex, and it can be difficult to mathematically predict flow patterns [7]. Several numerical simulation codes have been developed [8,9,10,11,12,13,14,15] to predict the flow behavior in twin-screw extrusions based on geometrical features, polymer properties, and processing conditions. Nevertheless, up to the present, no numerical model has been proposed to simulate and predict the flow behavior in OSEs.

This work investigated the octa-screw extrusion process, both experimentally and numerically, and assessed the degree of fill, residence time distribution (RTD), and temperature profile in the extruder. An intermeshing corotating parallel octa-screw kneading extruder was used for the experiments. High-density polyethylene (HDPE) and a heat-resistant masterbatch were selected as the materials for experiments. Meanwhile, a numerical code, based on a reconstructed flow analysis network (FAN) scheme, the Hele–Shaw flow model, finite element method (FEM), and a downwind pressure updating technique [14], was developed to calculate and estimate the resin profile in the octa-screw extrusion process. The flow velocities in both the axial and circumferential directions were considered in the proposed method. The numerical model was based on a 2.5 D FEM, which has conventionally been used in the injection molding simulation. Finally, a comparison study was also conducted in this research to determine the difference between octa-screw and twin-screw extrusions.

## 2. Materials and Methods

### 2.1. Materials

The polymeric material used in this study was HDPE (1300J, Prime Polymer Co., Tokyo, Japan) with a molecular weight of 3.4 × 106 Da. The EX-3052J-OIIB-MB33 model heat-resistance masterbatch purchased from Nippon Pigment Company Ltd., Tokyo, Japan, was used for the experiments.

### 2.2. Octa-Screw and Twin-Screw Extrusions

**Figure 1:**
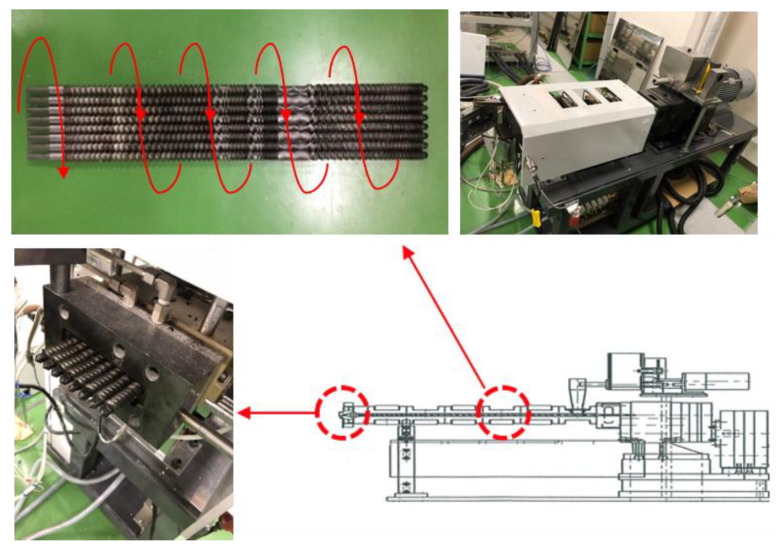
Photos of the octa-screw extruder used for the experiments.

An intermeshing corotating parallel octa-screw kneading extruder (WDR series 8, Technovel Co., Ltd., Tokyo, Japan) with a length over diameter (L/D) of 45 and a diameter of 15 mm was used for the experiments. As shown in Figure 1, the temperature settings of C1 and C2 on the two extruders near the hopper were 30 °C and 145 °C, respectively. Meanwhile, all other settings, such as C3–C5 on the extruders and H and D at the die exits, were maintained at 155 °C. The feed rate (Q) at the hopper was 0.73 kg/h, while two screw rotational speeds (N) of 100 and 200 rpm were used, corresponding to Q/N ratios of 0.0073 and 0.00365, respectively. For comparison, a twin-screw extruder (TSE; KZW15TW-30MG-NH, Technovel Co., Tokyo, Japan) with the same L/D and diameter as that of the OSE was also adopted.

### 2.3. Determining the Degree of Fill

OSEs and TSEs were first operated, until they reached a steady state, to determine the degree of fill in the multiscrew extruders [15]. The machines were then turned off, and the screws were removed from the barrels. After each kneading process, the weights of residual polymers on the screw elements were measured. The fill factor (*FF^e^*) that describes the degree of fill for each screw element was determined using the following equation:

(1) $$FF^{e}=\frac{wt^{emax}-wt^{e}}{wt^{emax}}$$

where *wt^emax^* is the maximum weight of the polymer when the element is completely filled, and *wt^e^* is the weight of the residual polymer on the screw element.

### 2.4. Assessment of Residence Time Distribution

The heat-resistance masterbatch of 12 mg was first blended with HDPE of 20 g via a dry mixer for subsequent extrusions to identify the RTD. The extruded resins at the die exit were collected every 30 s during extrusion. The collected samples were then analyzed using a thermogravimetric analyzer (TGA, TA Instruments QA-50, New Castle, DE, USA). The temperature of the TGA was programmed to rise from 30 °C to 500 °C at a constant heating rate of 5 °C/min. TGA tests were performed under an airflow of 60 mL/min. After the complete pyrolysis of the polymers, the amount of the remaining masterbatch was weighted with a balance of ±0.1 mg accuracy. RTD was then defined as the variation in weights of the residual masterbatch with time.

## 3. Numerical Analysis

A numerical code, based on 2.5 D FEM derived from the Hele–Shaw flow model [14], was used to calculate the resin profile in the OSE. The model has conventionally been used in the flow analysis of the injection molding process [16,17].

### 3.1. Formulation of Numerical Model

**Figure 2:**
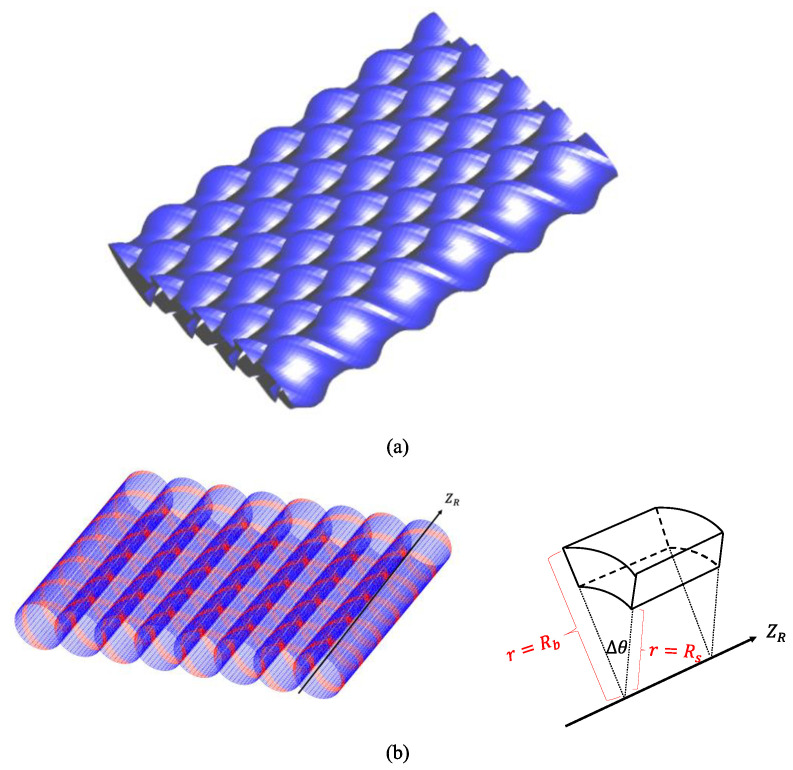
(**a**) Screw geometry and (**b**) 2.5 D elements for the octa-screw extruder.

**Figure 3:**
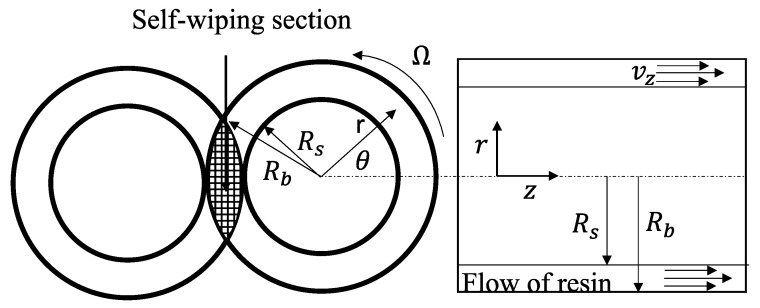
Schematic of the Hele–Shaw flow model.

The flow in the self-wiping section was neglected and the Hele–Shaw flow approximation was employed using a cylindrical coordinate (*r*, *θ*, *z*) system to simulate the flow pattern in an OSE (Figure 2 and Figure 3) at a low output for each screw. Assuming that the molten polymer is incompressible and acts as a purely viscous non-Newtonian fluid, adopting the Hele–Shaw approximation, the equation of continuity is reduced to:

(2) $$\frac{1}{r}\frac{\partial V_{z}}{\partial \theta }+\frac{\partial V_{z}}{\partial z}=0\;$$

Meanwhile, the equations of motion along the circumferential (*θ*) direction and axial (*z*) direction are

(3) $$\frac{1}{r^{2}}\frac{\partial }{\partial r}\left(r^{2}\eta \left(\frac{\partial V_{\theta }}{\partial r}-\frac{V_{\theta }}{r}\right)\right)=\frac{1}{r}\frac{\partial p}{\partial \theta }\;$$

and

(4) $$\frac{1}{r}\frac{\partial }{\partial r}\left(r\eta \frac{\partial V_{z}}{\partial r}\right)=\frac{\partial p}{\partial z}$$

where *p* is the pressure and *η* is the viscosity of the molten fluid. In this model, the screw is assumed to remain stationary and the barrel is considered to rotate relative to the screw with an angular velocity of Ω. Assuming the pressure gradient in the calculation element remains constant, then the flow velocities become:

(5) $$V_{\theta }=\frac{r}{2}\frac{\partial p}{\partial \theta }\left(\int_{R_{s}}^{r}\frac{1}{\eta r}dr-\left(\frac{\beta_{c}}{\gamma_{c}}\right)\int_{R_{s}}^{r}\frac{1}{\eta r^{3}}dr\right)+\left(\frac{\Omega r}{\gamma_{c}}\right)=\int_{R_{s}}^{r}\frac{1}{\eta r}dr$$

and

(6) $$V_{z}=\frac{1}{2}\frac{\partial p}{\partial z}\left(\int_{R_{s}}^{r}\frac{r}{\eta }dr-\frac{\alpha_{c}}{\beta_{c}}\int_{R_{s}}^{r}\frac{1}{\eta r}dr\right)\;$$

where *R_s_* is the screw radius. The strain rates with *θ_r_* and *z_r_* components can be, respectively, defined as:

(7) $${\dot{\gamma }}_{\theta r}\equiv r\frac{\partial }{\partial r}\left(\frac{V_{\theta }}{r}\right)=\frac{1}{2\eta }\left(\frac{\partial p}{\partial \theta }\right)\left(1-\frac{1}{r^{2}}\left(\frac{\beta_{c}}{\gamma_{c}}\right)\right)+\frac{1}{\eta r^{2}}\left(\frac{\Omega }{\gamma_{c}}\right)$$

and

(8) $${\dot{\gamma }}_{zr}\equiv \frac{\partial v}{\partial z}=\frac{1}{2\eta }\left(\frac{\partial p}{\partial z}\right)\left(r-\frac{1}{r}\left(\frac{\alpha_{c}}{\beta_{c}}\right)\right)\;$$

Additionally, the flow rates along the θ- and z-directions can be expressed, respectively, as:

(9) $$q_{\theta }\equiv \int_{R_{s}}^{R_{b}}v_{\theta }\mathrm{dr}=-\frac{1}{4}\frac{\partial p}{\partial \theta }\left(\alpha_{c}-\frac{\beta_{c}^{2}}{\gamma_{c}}\right)+\frac{\Omega }{2}\left(R_{b}^{2}-\frac{\beta_{c}}{\gamma_{c}}\right)$$

and

(10) $$q_{z}\equiv \int_{R_{s}}^{R_{b}}v_{z}\mathrm{rdr}=-\frac{1}{4}\frac{\partial p}{\partial z}\left(\delta_{c}-\frac{\alpha_{c}^{2}}{\beta_{c}}\right)\;$$

where *R_b_* is the barrel’s internal diameter, and *α_c_*, *β_c_*, *γ_c_*, and *δ_c_* denote integration constants along the r-direction [14].

Further, the steady-state heat transfer of the resin, barrel, and screw can be calculated using the equation of heat conduction. According to the Hele–Shaw approximation, the heat conduction through the θ- and z-directions can be neglected due to the dominant convection effects. The heat transfer equation can be expressed as

(11) $$\rho {\bar{C}}_{p}(\frac{V_{\theta }}{r}\frac{\partial T}{\partial \theta }+v_{z}\frac{\partial T}{\partial \theta })\equiv \lambda [\frac{1}{r}\frac{\partial }{\partial r}(r\frac{\partial T}{\partial \theta })]+\eta [r\frac{\partial }{\partial r}{\left(\frac{\partial v_{z}}{\partial r}\right)}^{2}+{\left(\frac{\partial v_{z}}{\partial r}\right)}^{2}]$$

where *T* is the absolute temperature, *ρ* is the density, *λ* is the thermal conductivity, and *C_p_* is the heat capacity.

The 2.5 D finite element code was then developed based on the above formulations. Numerical analysis was conducted to simulate the flow velocity, strain rate, flow rate, and temperature distribution in an OSE and a TSE with the assigned boundary conditions.

### 3.2. Estimating the Fill Factor

The finite element calculation was first performed on a hypothetical fully filled situation to estimate the degree of filling material. The pressure of each element was calculated by obtaining the pressure gradient necessary to determine the pressure distribution within the elements in the case of the unfilled screw. As the flow behavior depends on the pressure gradient, the distribution of head pressure can be calculated using the downwind pressure update scheme [14] given by:

(12) $$p\left(z-\Delta z,\theta -\Delta \theta \right)=p\left(z,\theta \right)-\frac{\partial p}{\partial z}\Delta z-\frac{\partial p}{\partial \theta }\Delta \theta$$

This calculation is performed along the axial direction opposite from the hopper. The pressure gradient in the downstream element is used preferentially, and any element that is calculated to have a negative or zero pressure is decided to be unfilled.

The fill factor, *FF^z^*, for finite elements constituting the plane perpendicular to the *z*-axis of the screw can be defined as:

(13) $$f^{Z}=\frac{Q_{\mathrm{ext}}}{\sum^{\;}Q_{d}^{Z}}$$

The fill factor of each element, *FF^e^*, is determined by recalculating the pressure in the filled state *FF^e^* = 1 and in the unfilled state *FF^e^* = 0. The volume-weighted average of the *FF^av^* can be evaluated using the following equation:

(14) $$f^{av}=\frac{\sum^{\;}V^{e}f^{e}}{\sum^{\;}V^{e}}$$

where *V^e^* is the volume of each element.

## 4. Results and Discussion

### 4.1. Degree of Fill

**Figure 4:**
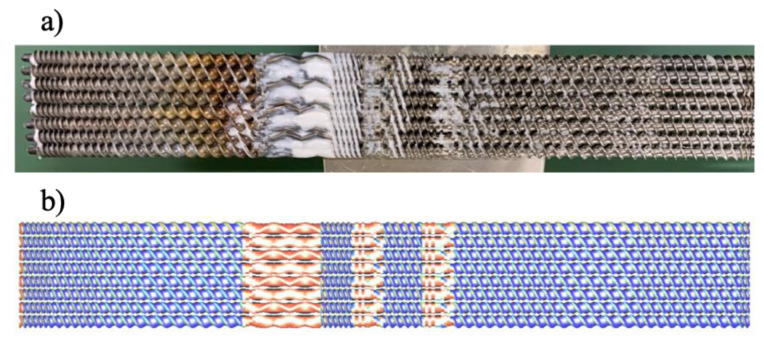
(**a**) Photograph of the fill of polymer resins on the retrieved screws of an octa-screw extruder (from right to left: hopper side to exit side), and (**b**) the simulated degree of fill.

**Figure 5:**
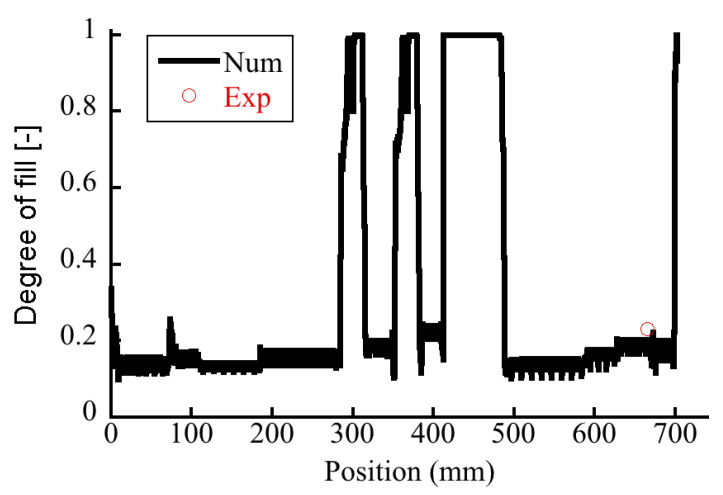
Degree of fill along the screw in the octa-screw extruder.

It is essential to understand the extent of partial filling of the flight screw, i.e., degree of fill, which is an operational variable of the OSE. Taki et al. [18] found that the degree of fill was inversely proportional to the rotational speed and proportional to the feed rate. Robinson and Cleary [19] studied the effect of degree of fill on the transport and mixing behavior of a corotating TSE and found that a fill level of 50% produces the highest mixing rates for the screw elements. The degree of fill for the OSE was determined. Figure 4a shows the experimental photo of retrieved screws from the octo-screw extruder, suggesting that the extruder was operated under starved conditions. Figure 5 records the degree of fill in the extruder. In most of the areas along the screws, the degree of fill was merely 20%. Fully filled zones could only be found at 300, 360, and 420–490 mm along the screw, corresponding to the kneading block I, kneading block II, and rotor element section, respectively.

**Figure 6:**
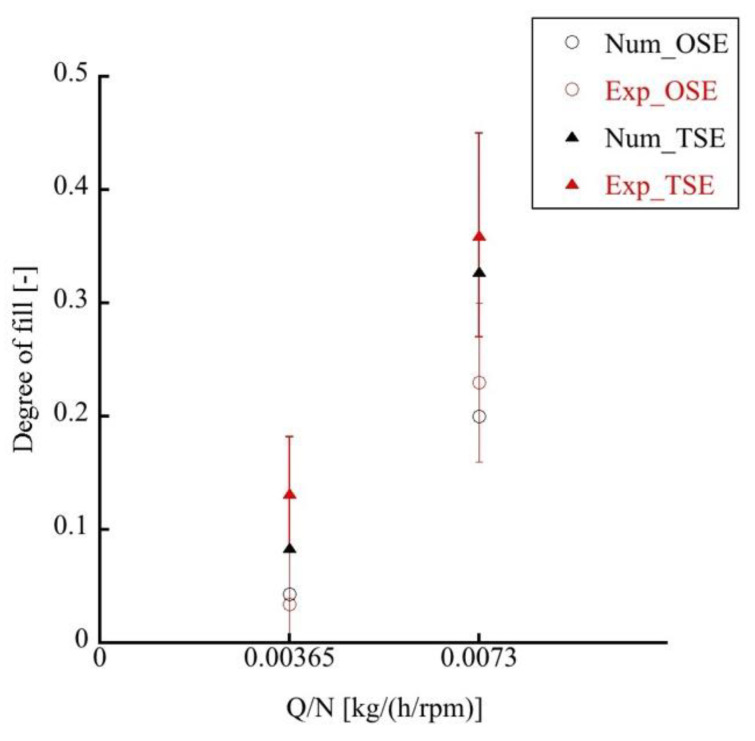
Measured and calculated degrees of fill in twin- and octa-screw extrusions.

The influence of the extrusion rate on the degree of fill was also examined. The measured data in Figure 6 showed that the degree of fill for the OSE increased from 4% to 23% when the Q/N ratio was increased from 0.00365 (200 rpm) to 0.0073 (100 rpm). Meanwhile, the degree of fill for the TSE raised from 13% to 36% when the Q/N ratio was increased from 0.00365 to 0.0073. Clearly, the OSE displayed a lower degree of fill than that of the TSE. The material feeding rates for TSEs and OSEs were the same. As the octa-screw had a larger transport capability, it possessed a lower degree of fill during extrusion.

The degree of fill was also estimated numerically. The degree of fill was calculated by dividing the sum of the filled elements in the FEM and the total capacity of the screws (Equation (14)). Figure 4b shows the predicted degree of fill along the axis of the OSE. The numerical results suggest fully filled zones at the kneading block I, kneading block II, and rotor element section. Additionally, the calculated degrees of fill or TSEs and OSEs were compared to the measured data at the location denoted by a red circle in Figure 5. The simulated degrees of fill in Figure 6 showed good agreement with the experimental data under both high and low Q/N operation conditions, which testified the validity of the numerical code developed in this study.

### 4.2. Temperature Distribution

**Figure 7:**
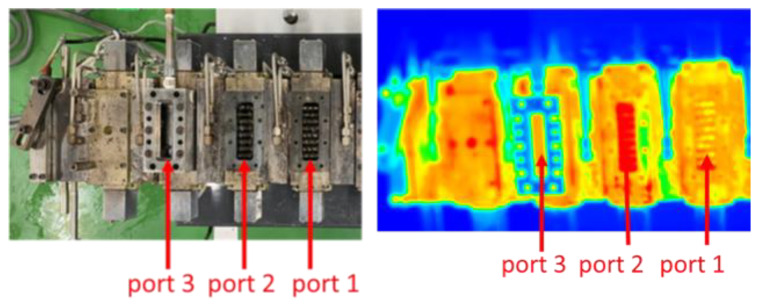
Thermal image of temperature distribution in the octa-screw extruder.

**Figure 8:**
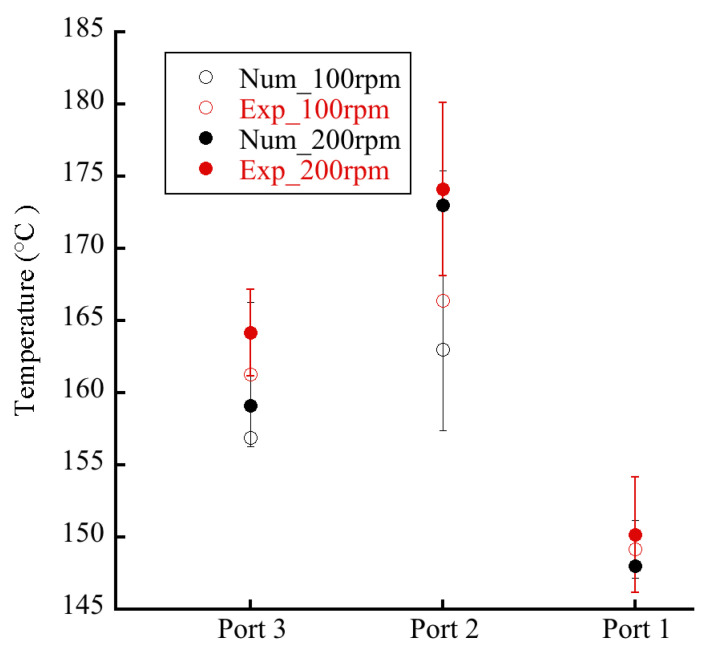
Measured and calculated temperature distributions in an octa-screw extruder.

Extrusion is one of the most used processes for producing polymeric materials, and the thermal homogeneity of the process has paramount importance for the manufacture of high-quality extruded products [20,21]. Hence, accurate process thermal monitoring and control are important for product quality control [22]. Figure 7 illustrates the measured thermal image of an OSE, and Figure 8 shows the measured temperatures at ports 1, 2, and 3. The empirical results suggest that a higher temperature could be found at the zone near port 2, corresponding to the section of kneading blocks where extensive compounding was achieved. Additionally, as expected, the extruder exhibited a higher temperature distribution at a rotational speed of 200 rpm (Q/N = 0.00365) than at 100 rpm (Q/N = 0.0073).

**Figure 9:**
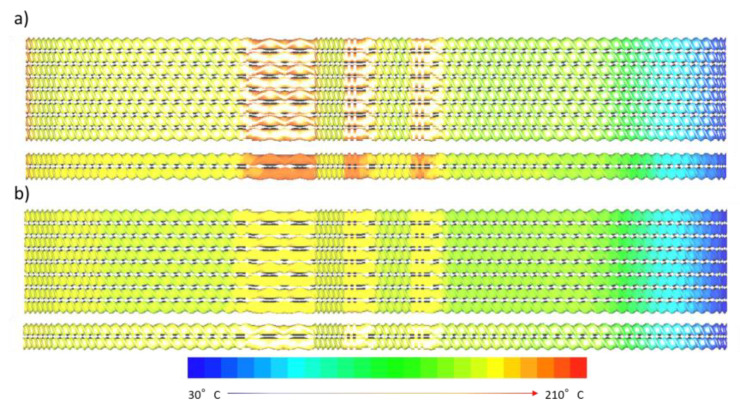
Calculated temperature distribution in octa- and twin-screw extruders operated at Q/N ratios of (**a**) 0.00365 and (**b**) 0.0073.

The influence of extrusion speed on the temperature profiles was also numerically simulated. Figure 9a,b show the calculated temperature distribution in OSEs and TSEs, subject to Q/N ratios of 0.00365 (200 rpm) and 0.0073 (100 rpm), respectively. A greater extrusion rate was calculated to generate higher temperature distributions in the extruders. Additionally, the simulated results in Figure 9 also suggested that the OSE displayed a lower temperature distribution than that of the TSE during the extrusion process, which demonstrates the advantages of the OSE in terms of superior mixing capability with minimized thermal degradation of polymeric materials in the extrusion process.

The simulated temperature was also compared with the experimental data to check the validity. The results in Figure 8 showed that the numerical simulation agreed well with the experimental data, further demonstrating the effectiveness of the numerical model proposed in this study.

### 4.3. Residence Time Distribution

**Figure 10:**
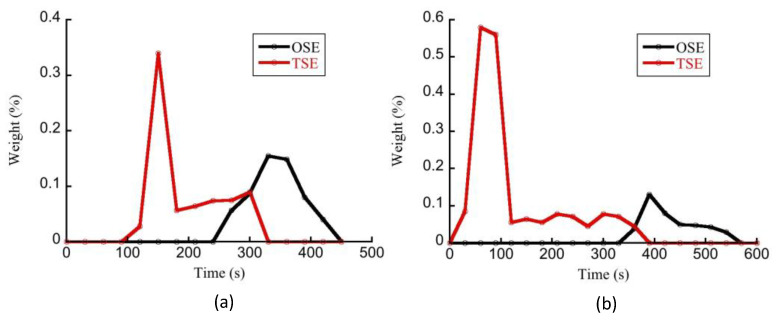
Residence time distribution along the screws of OSE and TSE operated at (**a**) Q/N = 0.0035 (200 rpm) and (**b**) Q/N = 0.0073 (100 rpm).

The comprehension of the RTD is critical for polymer extrusion because it is related to the level of polymer degradation and special processes such as reactive extrusion [23]. The thermal history of a polymer material can affect the final product quality and is closely related to the RTD and temperature distribution in the octa-screw extrusion process. It is important to know the resin distribution over the screws to determine the RTD. For that reason, the resin distribution is a key factor for octa-screw extrusion depending on the screw design, feed rate, screw speed, and rheological properties of the resin. For different applications, such as dispersing, blending, and polymerization, the RTD tool in an extruder has great importance in determining the optimal processing conditions [24]. This is particularly true in the case of OSEs where the complex geometry has so far prevented us from reaching the same state of knowledge that we have about TSEs. Figure 10a shows the measured RTD of OSEs and TSEs at a higher extrusion rate (200 rpm, Q/N = 0.00365), while Figure 10b displays the RTD operated at a lower extrusion rate (100 rpm, Q/N = 0.0073). The residence time in Figure 10b was longer because the screw rpm was decreased, pushing out the melt slower. An experiment doubling Q at the same N will show different results, displaying decreased residence time. Additionally, the experimental results suggest that the OSE exhibited a narrower RTD than the TSE did. The residence time in the OSE with much more free volume was obviously longer than that in TSE, and that would result in a better extrudate quality but higher melt temperature.

Finally, it should be noted that the OSE is similar to the ring extruder (or planetary roller extruder), except that the screws are arranged on a flat plane. It has been reported that the planetary roller extruder is one of the most successful multi-screw extruders in pelleting and compounding powdered paint and recyclate [25]. Schmidt et al. compared the TSE and the ring extruder in terms of their performance on granulation/extrusion and spheronization [26,27]. They proposed that excluding the influence of the spheronization process, high differences were noted in the quality of the extrudates prepared via these two extruders. With the ring die press, an additional influence of the particle size could be observed. Different extruders also applied distinct mechanical stress on the extrudate, and, in turn, affected the network structure of the microcrystalline cellulose gel. In this study, we made a comparison between the OSE and TSE, both experimentally and numerically. Whether the OSE performs better than the ring extruder or not requires additional works for verification. These will be the topics of our future research.

## 5. Conclusions

We experimentally and numerically investigated the octa-screw extrusion process and assessed the degree of fill, RTD, and temperature profile in the extruder. An intermeshing corotating parallel octa-screw kneading extruder was employed for the experiments. Meanwhile, a numerical code based on a reconstructed FAN scheme, the Hele–Shaw flow model, FEM, and a downwind pressure updating technique was developed to simulate the octa-screw and twin-screw extrusion processes. The empirical outcomes suggest that octa-screw extrusion exhibited a narrower resin distribution time than the twin-screw extrusion. The simulated results also showed that the OSE displays a lower temperature distribution than the TSE did during the extrusion process. This proves the advantages of the OSE in terms of superior mixing capability with minimized thermal degradation of polymeric materials in the extrusion process. The results of the simulation were also found to be in good agreement with experimental measurements. Experimental and numerical investigations of an OSE enable detailed comprehension and visualization of resin distribution over the length of the OSE, thus enhancing their capability in terms of process optimization.

## Data Availability

The data presented in this study are available upon request from the corresponding author.
